# Supplementary material for: Comparing Heterologous and Homologous COVID-19 Vaccination: A Longitudinal Study of Antibody Decay
Source: Viruses. 2023 May 13;15(5):1162. doi: 10.3390/v15051162 (PMC10222288; doi:10.3390/v15051162)
Supplement: Supplementary file 1 [file viruses-15-01162-s001.zip › viruses-2384245-Supplementary.pdf]

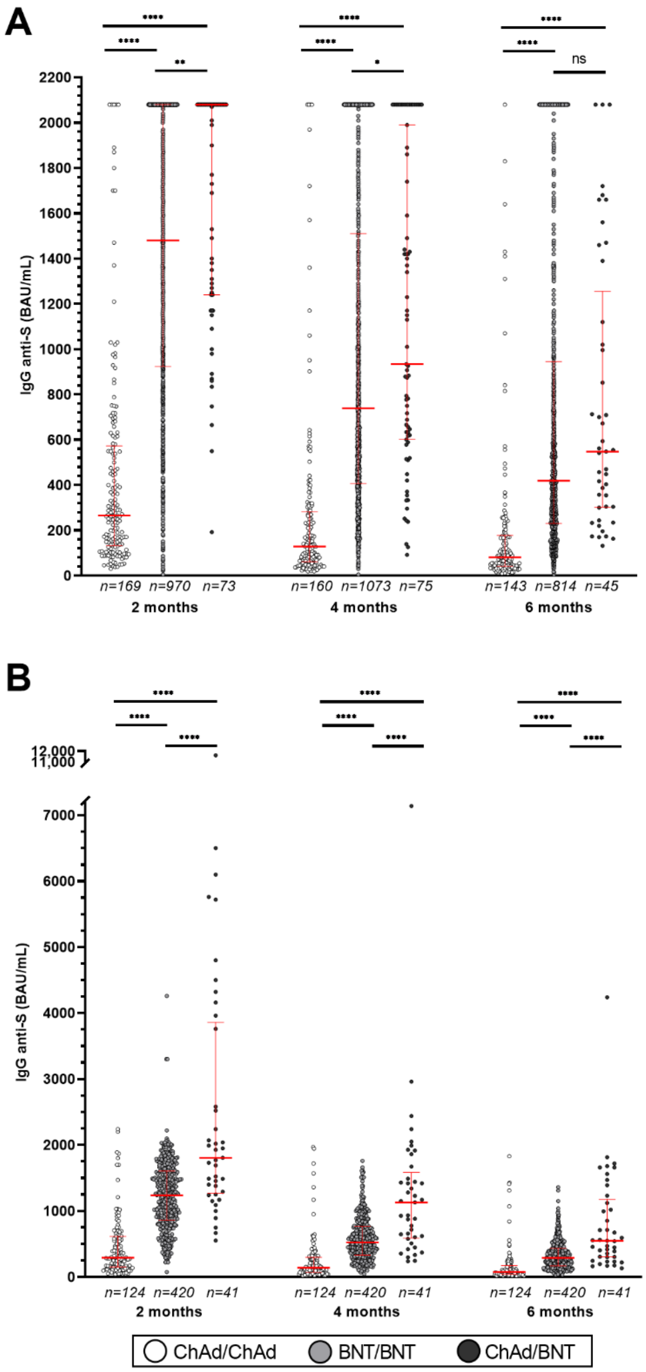

Figure S1. Inter-group comparison of SARS-CoV-2 anti-trimeric Spike protein IgG among three different groups of immunized subjects at two, four and six months after vaccination. (**A**) All subjects (different number of patients reported in the figure). (**B**) Subgroup of subjects who completed the post-vaccination follow-up and whose blood samples showed a descending phase of the anti-S IgG concentration (n = 585). Each dot represents a sample and red lines represent the median and interquartile range. Kruskal Wallis test with Dunn's post hoc multiple comparisons. * $p \le 0.05$; ** $p \le 0.01$; **** $p \le 0.0001$; ns = not statistically significant.

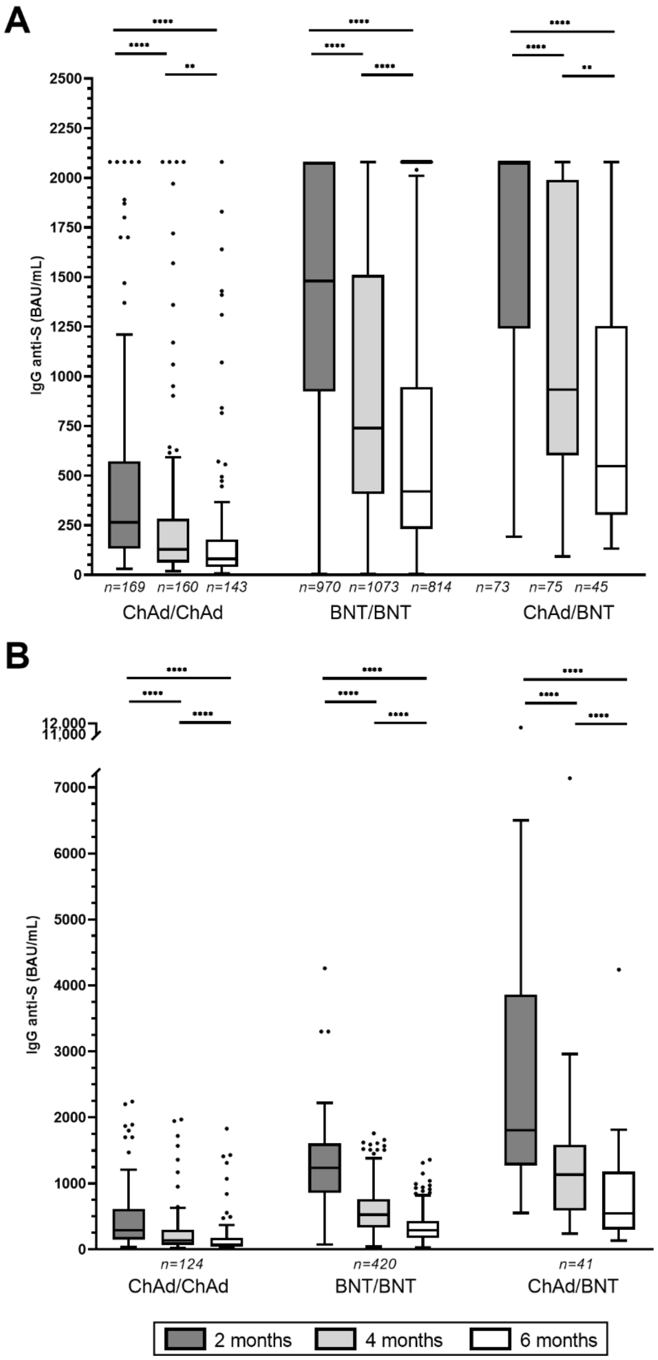

Figure S2. Intra-group comparison of anti-trimeric SARS-CoV-2 Spike protein IgG among three different groups of immunized subjects at two, four and six months after vaccination. (**A**) All subjects. (**B**) Subgroup of subjects who completed the post-vaccination follow-up and whose blood samples showed a descending phase of the anti-S IgG concentration. The box represents the interquartile range and the whiskers are extending to the lowest and highest value within 1.5 times the interquartile range from the hinges, respectively. Unpaired Kruskal-Wallis (A) or paired Friedman's (B) tests with Dunn's multiple comparison were used for *post hoc* analysis. ** $p \leq 0.01$; **** $p \leq 0.0001$

Table S1. Results of anti- nucleocapsid (N) antibodies testing.

Vaccine schedule	Anti-N	Number of positive serum samples at			Total positive subjects per antibody class	Total positive subjects per vaccine schedule
		2-months	4-months	6-months		
ChAd/ChAd *n* = 120 (70%)	IgM	5	6	4	6 (5%)	11 (9%)
	IgG	3	4	6	6 (5%)	
BNT/BNT *n* = 10 (6%)	IgM	0	1	0	1 (10%)	2 (20%)
	IgG	0	0	1	1 (10%)	
ChAd/BNT n = 41 (24%)	IgM	2	4	3	4 (10%)	4 (10%)
	IgG	1	1	1	1 (2.4%)	
Total	IgM	7	11	7	11	17 (10%)
	IgG	4	5	8	8	

Two subjects (one in ChAd/ChAd and one in ChAd/BNT) presented both anti-N IgM and IgG positive during the analysis.

Table S2. Results of analysis of covariance (ANCOVA) in a group of 585 vaccinated subjects: anti-S IgG antibody level at 2-4-6-months after vaccination and kinetic parameters, corrected for vaccine schedule, sex and age.

A. Anti-S IgG antibody level at 2-months

Source	Type III Sum of Squares	df	Mean Square	F	Sig.	Noncent. Parameter	Observed Power[b]
Corrected Model	151,431,734.933a	4	37,857,933.733	68.049	≤ 0.0001	272.198	1.000
Intercept	41,371,578.444	1	41,371,578.444	74.365	≤ 0.0001	74.365	1.000
**Vaccine schedule**	1,464,741,41.058	2	73,237,070.529	131.643	**≤ 0.0001**	263.286	1.000
**Sex**	1,102,293.408	1	1,102,293.408	1.981	0.160	1.981	0.290
**Age**	380,65.957	1	38,065.957	0.068	0.794	0.068	0.058
Error	322,671,533.880	580	556,330.231				
Total	1,261,283,908.820	585					
Corrected Total	474,103,268.813	584					

[a.] R-Squared = 0.319 (Adjusted R Squared = 0.315)

[b.] Computed using alpha = 0.05

B. Anti-S IgG antibody level at 4-months

Source	Type III Sum of Squares	df	Mean Square	F	Sig.	Noncent. Parameter	Observed Power[b]
Corrected Model	32,505,501.581[a]	4	8,126,375.395	41.972	≤ 0.0001	167.886	1.000
Intercept	11,420,057.657	1	11,420,057.657	58.983	≤ 0.0001	58.983	1.000
**Vaccine schedule**	31,436,564.133	2	15,718,282.067	81.183	**≤ 0.0001**	162.365	1.000
**Sex**	1000.950	1	1000.950	0.005	0.943	0.005	0.051
**Age**	101,488.142	1	101,488.142	0.524	0.469	0.524	0.112
Error	112,297,478.541	580	193,616.342				
Total	327,113,557.610	585					
Corrected Total	144,802,980.122	584					

[a.] R-Squared = 0.224 (Adjusted R Squared = 0.219)

[b.] Computed using alpha = 0.05

C. Anti-S IgG antibody level at 6-months

Source	Type III Sum of Squares	df	Mean Square	F	Sig.	Noncent. Parameter	Observed Power[b]
Corrected Model	12,465,491.921[a]	4	3,116,372.980	35.047	≤ 0.0001	140.190	1.000
Intercept	4,128,564.236	1	4,128,564.236	46.431	≤ 0.0001	46.431	1.000
**Vaccine schedule**	12,162,313.458	2	6,081,156.729	68.390	**≤ 0.0001**	136.780	1.000
**Sex**	27,630.981	1	27,630.981	0.311	0.577	0.311	0.086
**Age**	20,518.706	1	20,518.706	0.231	0.631	0.231	0.077
Error	51,572,936.260	580	88,918.856				
Total	127,148,437.010	585					
Corrected Total	64,038,428.181	584					

[a.] R-Squared = 0.195 (Adjusted R Squared = 0.189)

[b.] Computed using alpha = 0.05

D. Time to negativization

Source	Type III Sum of Squares	df	Mean Square	F	Sig.	Noncent. Parameter	Observed Power[b]
Corrected Model	684,571.570[a]	4	171,142.892	3.637	0.006	14.546	0.878
Intercept	3,080,906.277	1	3,080,906.277	65.465	≤ 0.0001	65.465	1.000
**Vaccine schedule**	593,397.068	2	296,698.534	6.304	**0.002**	12.609	0.897
**Sex**	394.441	1	394.441	0.008	0.927	0.008	0.051
**Age**	46,289.855	1	46,289.855	0.984	0.322	0.984	0.168
Error	27,296,038.306	580	47,062.135				
Total	91,668,981.105	585					
Corrected Total	27,980,609.876	584					

a. R-Squared = 0.024 (Adjusted R Squared = 0.018)

b. Computed using alpha = 0.05

E. Rate constant k

Source	Type III Sum of Squares	df	Mean Square	F	Sig.	Noncent. Parameter	Observed Power[b]
Corrected Model	0.000[a]	4	684,500	5.485	≤ 0.0001	21.942	0.976
Intercept	0.002	1	0.002	162.896	≤ 0.0001	162.896	1.000
**Vaccine schedule**	0.000	2	0.000	10.062	**≤ 0.0001**	20.124	0.985
**Sex**	102,000	1	102,000	0.817	0.366	0.817	0.147
**Age**	8,315,000	1	8,315,000	0.666	0.415	0.666	0.129
Error	0.007	580	124,800				
Total	0.083	585					
Corrected Total	0.008	584					

a. R-Squared = 0.036 (Adjusted R Squared = 0.030)

b. Computed using alpha = 0.05

F. Half-life

Source	Type III Sum of Squares	df	Mean Square	F	Sig.	Noncent. Parameter	Observed Power[b]
Corrected Model	39,895.938[a]	4	9973.984	7.545	≤ 0.0001	30.182	0.997
Intercept	124,809.576	1	124,809.576	94.420	≤ 0.0001	94.420	1.000
**Vaccine schedule**	38,151.051	2	19,075.525	14.431	**≤ 0.0001**	28.862	0.999
**Sex**	497.417	1	497.417	0.376	0.540	0.376	0.094
**Age**	755.010	1	755.010	0.571	0.450	0.571	0.117
Error	766,675.226	580	1321.854				
Total	3,663,915.688	585					
Corrected Total	806,571.164	584					

a. R-Squared = 0.049 (Adjusted R Squared = 0.043)

b. Computed using alpha = 0.05

Table S3. Characteristics of a group of 171 vaccinated subjects included in a second ANCOVA analysis.

Factor	*n* = 120 (70%)	*n* = 10 (6%)	*n* = 41 (24%)
Vaccine schedule	ChAd/ChAd;	BNT/BNT	ChAd/BNT
Sex (Male)	54 (45%)	3 (30%)	19 (46%)
Age	56 (48-62)	47 (41-62)	53 (41-58)
Smoking	12 (10%)	0 (0%)	9 (22%)
BMI	23.72 (21.81-27.2)	23.18 (21.12-26.34)	23.42 (21.83-25.53)

Table S4. Results of analysis of covariance (ANCOVA) in a subgroup of 171 subjects from the university staff: anti-S IgG antibody level at 2-4-6-months after vaccination and kinetic parameters, corrected for several factors

A. Anti-S IgG antibody level at 2-months

Source	Type III Sum of Squares	df	Mean Square	F	Sig.	Noncent. Parameter	Observed Power[b]
Corrected Model	165,151,961.449a	6	27,525,326.908	20.936	0.000	125.618	1.000
Intercept	2,561,991.687	1	2,561,991.687	1.949	0.165	1.949	0.284
**Vaccine schedule**	160,998,865.708	2	80,499,432.854	61.229	**≤ 0.0001**	122.459	1.000
**Sex**	1,490,894.146	1	1,490,894.146	1.134	0.288	1.134	0.185
**Age**	3,579,132.862	1	3,579,132.862	2.722	0.101	2.722	0.375
**Smoking**	75,454.456	1	75,454.456	0.057	0.811	0.057	0.057
**BMI**	51,662.603	1	51,662.603	0.039	0.843	0.039	0.054
Error	215,613,936.891	164	1,314,719.127				
Total	579,717,335.820	171					
Corrected Total	380,765,898.340	170					

[a.] R-Squared = 0.434 (Adjusted R Squared = 0.413)

[b.] Computed using alpha = 0.05

B. Anti-S IgG antibody level at 4-months

Source	Type III Sum of Squares	df	Mean Square	F	Sig.	Noncent. Parameter	Observed Power[b]
Corrected Model	37,595,736.211a	6	6,265,956.035	15.112	0.000	90.673	1.000
Intercept	2,392,903.471	1	2,392,903.471	5.771	0.017	5.771	0.666
**Vaccine schedule**	35,376,805.736	2	17,688,402.868	42.661	**≤ 0.0001**	85.322	1.000
**Sex**	159,833.659	1	159,833.659	0.385	0.536	0.385	0.095
**Age**	985,984.631	1	985,984.631	2.378	0.125	2.378	0.335
**BMI**	31,046.911	1	31,046.911	0.075	0.785	0.075	0.059
Error	1,196,695.342	1	1,196,695.342	2.886	0.091	2.886	0.393
Total	67,999,148.559	164	414,628.955				
Corrected Total	157,812,490.210	171					

[a.] R-Squared = 0.356 (Adjusted R Squared = 0.332)

[b.] Computed using alpha = 0.05

C. Anti-S IgG antibody level at 6-months

Source	Type III Sum of Squares	df	Mean Square	F	Sig.	Noncent. Parameter	Observed Power[b]
Corrected Model	15,039,404.072a	6	2,506,567.345	12.376	0.000	74.256	1.000
Intercept	1,162,256.097	1	1,162,256.097	5.739	0.018	5.739	0.663
**Vaccine schedule**	13,662,918.350	2	6,831,459.175	33.730	**≤ 0.0001**	67.460	1.000
**Sex**	67,199.259	1	67,199.259	0.332	0.565	0.332	0.088
**Age**	614,018.290	1	614,018.290	3.032	0.084	3.032	0.410
**Smoking**	4996.125	1	4996.125	0.025	0.875	0.025	0.053
**BMI**	835,308.619	1	835,308.619	4.124	**0.044**	4.124	0.524
Error	33,215,627.284	164	202,534.313				

Total	69,851,196.760	171
Corrected Total	48,255,031.356	170

[a.] R-Squared = 0.312 (Adjusted R Squared = 0.286)

[b.] Computed using alpha = 0.05

D. Time to negativization

Source	Type III Sum of Squares	df	Mean Square	F	Sig.	Noncent. Parameter	Observed Power[b]
Corrected Model	1,619,158.952a	6	269,859.825	2.346	0.034	14.074	0.797
Intercept	1,710,566.500	1	1,710,566.500	14.869	0.000	14.869	0.969
**Vaccine schedule**	627,127.657	2	313,563.828	2.726	0.068	5.451	0.533
**Sex**	193,019.049	1	193,019.049	1.678	0.197	1.678	0.251
**Age**	17.881	1	17.881	0.000	0.990	0.000	0.050
**Smoking**	104.704	1	104.704	0.001	0.976	0.001	0.050
**BMI**	819,865.276	1	819,865.276	7.126	**0.008**	7.126	0.756
Error	18,867,575.250	164	115,046.191				
Total	40,181,716.806	171					
Corrected Total	20,486,734.202	170					

a. R-Squared = 0.079 (Adjusted R Squared = 0.045)

[b.] Computed using alpha = 0.05

E. Rate constant k

Source	Type III Sum of Squares	df	Mean Square	F	Sig.	Noncent. Parameter	Observed Power[b]
Corrected Model	0.000a	6	276,000	1.993	0.070	11.955	0.717
Intercept	720,100	1	720,100	5.199	0.024	5.199	0.621
**Vaccine schedule**	280,300	2	140,200	1.012	0.366	2.024	0.224
**Sex**	790,200	1	7,902,005	5.705	**0.018**	5.705	0.661
**Age**	1,261,000	1	1,261,000	0.091	0.763	0.091	0.060
**Smoking**	384,000,000	1	384,000,000	0.003	0.958	0.003	0.050
**BMI**	967,000	1	967,000	6.981	**0.009**	6.981	0.748
Error	0.002	164	138,500				
Total	0.020	171					
Corrected Total	0.002	170					

[a.] R-Squared = 0.068 (Adjusted R Squared = 0.034)

[b.] Computed using alpha = 0.05

F. Half-life

Source	Type III Sum of Squares	df	Mean Square	F	Sig.	Noncent. Parameter	Observed Power[b]
Corrected Model	33,281.256a	6	5546.876	1.851	0.092	11.109	0.679
Intercept	61,033.300	1	61,033.300	20.372	0.000	20.372	0.994
**Vaccine schedule**	7411.899	2	3705.950	1.237	0.293	2.474	0.267
**Sex**	9177.451	1	9177.451	3.063	0.082	3.063	0.413
**Age**	491.755	1	491.755	0.164	0.686	0.164	0.069
**Smoking**	304.992	1	304.992	0.102	0.750	0.102	0.062

**BMI**	21,723.987	1	21,723.987	7.251	**0.008**	7.251	0.763
Error	491,326.102	164	2995.891				
Total	1,690,509.908	171					
Corrected Total	524,607.358	170					

[a.] R-Squared = 0.063 (Adjusted R Squared = 0.029)

[b.] Computed using alpha = 0.05
